# Effects of Static Stretching on the Blood Circulation of Human Tendon In Vivo

**DOI:** 10.1155/2024/4413113

**Published:** 2024-04-10

**Authors:** Ayaka Yasuda, Keitaro Kubo

**Affiliations:** Department of Life Science (Sports Sciences), The University of Tokyo, Komaba 3-8-1, Meguro-Ku, Tokyo 153-8902, Japan

## Abstract

The purpose of this study is to compare the effects of 2- and 5 min of static stretching protocols on the changes in blood circulation of tendon (as well as muscle) and heart rate. Twelve healthy males (age: 26.2 ± 9.1 yrs) volunteered for this study. Before, during stretching, during the recovery period (30 min), blood circulation (oxyhemoglobin; oxy, deoxyhemoglobin; deoxy, blood volume; THb, oxygen saturation; StO_2_) of the Achilles tendon and medial gastrocnemius muscle were measured using red laser lights and near-infrared spectroscopy. In addition, heart rate was measured during the experimental period. For 2- and 5 min of static stretching, oxy, deoxy, THb, and StO_2_ of the tendon did not change during or after stretching. Regarding muscle blood circulation, oxy and StO_2_ decreased, and deoxy and THb increased during 2- and 5 min of static stretching but returned immediately upon completion. In addition, heart rate significantly reduced during and after stretching, whereas the changes in blood volume of tendon and muscle during stretching were not associated with those in heart rate (except for the relationship between tendon THb and heart rate in 2 min of static stretching). These results suggest that static stretching showed no change in tendon blood circulation, although muscle blood circulation during stretching was altered. In addition, significant heart rate reduction with static stretching was not associated with changes in tendon and muscle blood circulation.

## 1. Introduction

Stretching is implemented not only to improve flexibility but also to promote muscle blood circulation. Several studies using ultrasound Doppler and near-infrared spectroscopy reported that muscle blood flow and blood volume decreased during stretching due to reduced vessel diameter and increased intramuscular pressure, and reactive hyperemia was seen after stretching [1–7]. More recently, Matsuo et al. [4] examined changes in muscle blood volume with static stretching of five different durations (20 s, 1, 2, 5, and 10 min) and found no difference in muscle blood volume increase after stretching for more than 2 min. Their result indicated that the minimum time for static stretching to maintain an increase in muscle blood volume after stretching was 2 min.

In athletic and medical fields, various therapies (e.g., hyperthermia and acupuncture) have been used to repair tendon disorders. Increased blood circulation in tendons is thought to be one possible mechanism for the repair of damaged tendons [8, 9]. Previous studies showed that blood circulation of tendons is enhanced by hyperthermia, acupuncture, hyperbaric oxygen therapy, and repeated eccentric contractions [10–13]. However, as the oxidative metabolic rate of tendons is meager [14, 15], tendons are thought to respond more slowly to various stimuli than muscles. We previously reported that tendon blood volume continued to increase up to 35 min due to 60 min of heating stimulation, whereas muscle blood volume showed no increase after 20 min [13]. Therefore, the minimum time for increasing the tendon blood volume may be longer than the minimum time for increasing the muscle blood volume presented by Matsuo et al. [4]. If tendon blood circulation is increased after stretching, stretching may be useful as a maintenance method to increase tendon blood circulation because it does not require special tools.

Several studies demonstrated decreased heart rate and increased parasympathetic nervous system activity with stretching [16–18]. On the other hand, Yamato et al. [7] reported that the increase in muscle blood flow by static stretching may be due to a local hemodynamic response (possibly through endothelial function) but not a central hemodynamic response (e.g., heart rate and blood pressure). However, to our knowledge, no direct relationship between stretching-induced changes in muscle blood flow and central hemodynamic response has been examined. Regarding tendon blood circulation, our previous studies showed that the changes in tendon blood volume during the recovery period after acupuncture and acupressure stimulation were significantly associated with the change in heart rate [11, 19]. Thus, if stretching causes changes in tendon (and muscle) blood circulation, the changes in muscle and tendon blood circulation would be related to the changes in the heart rate.

In the present study, we aimed to compare the effects of 2- and 5 min of static stretching on the changes in tendon blood circulation and heart rate variability, whereas no difference was observed in the increase in muscle blood volume after stretching between 2- and 5 min of static stretching [4]. Unlike muscle blood circulation, we hypothesized the change in tendon blood circulation after 5 min of static stretching to be greater than after 2 min of static stretching. Furthermore, we expected that stretching-induced changes in blood circulation of tendon (as well as muscle) would be associated with the changes in heart rate.

## 2. Materials and Methods

### 2.1. Participants

Twelve healthy males (age: 26.2 ± 9.1 yrs, height: 172.2 ± 7.6 cm, weight: 67.8 ± 10.0 kg, mean ± SD) volunteered for this study. They were either sedentary or slightly to moderately active men, but none had participated in a resistance training regimen for at least a year before the test. They were informed about the purposes of the investigation and the method to be utilized. Before starting the study, written informed consent was acquired. The Ethics Committee for Human Experiments, Department of Life Science (Sports Sciences), the University of Tokyo approved the present study.

### 2.2. Static Stretching

Static stretching of two different durations (2 and 5 min) was performed by the participant's right lower leg in the prone position. Each participant took two exams on separate days, with a minimum of 1 week between sessions. The order of the two experimental tests was randomized for each participant. The foot was securely connected to a foot plate that was attached to the lever arm of a dynamometer, and the ankle joint was adjusted to the neutral ankle position (0 degrees) with the knee joint at full extension (Applied Office, Tokyo, Japan). Participants rested comfortably in the prone position during the baseline 20 min rest period. After that, the dynamometer's platform, which was fastened to the participant's foot sole, was adjusted to 27 degrees of dorsiflexion and maintained there for 2 or 5 min. Participants were instructed to remain completely relaxed and not to provide any deliberate resistance during the stretching process. The following measurements (see below) were performed continuously before, during, and after static stretching.

### 2.3. Blood Circulation of Muscle and Tendon

During the experiment period, the total hemoglobin (corresponding to blood volume; THb) and oxygen saturation (StO_2_) of the medial gastrocnemius muscle and Achilles tendon were measured using near-infrared spectroscopy (BOM-L1TR, Omega Wave, Japan) and red laser lights (BOM-L1TRSF, Omega Wave, Japan). Regarding the THb and StO_2_ of the muscle, a probe was positioned in the middle of the muscle between the lateral malleous of the fibula and the lateral condyle of the tibia. This device determines the relative tissue levels of oxyhemoglobin (Oxy), deoxyhemoglobin (Deoxy), and THb using three laser diodes (780, 810, and 830 nm). The distance between the light source and the photodetector was 3 cm. Regarding the THb and StO_2_ of the tendon, a probe was positioned at 20 mm proximal from the calcaneus. This device determines the relative tissue levels of THb, deoxy, and oxy using three red laser beams (635, 650, and 690 nm). The distance between the light source and the photodetector was 5 mm. By changing the location of the two detectors, the measured variables at a particular tissue depth (measurement depth of 3–5 mm) could be determined. We previously described the details of the principles of this instrument (e.g., Kubo et al. [15]).

In this study, the Oxy, Deoxy, and THb units were expressed as *μ*mol/l, although the measured variables did not represent the actual physical volume. StO_2_ was calculated using the formula: StO_2_ (%) = 100·Oxy·THb^−1^. The mean values over a given duration (last 10 min of resting before stretching, every 1 min during stretching, and every 5 min during the recovery period) were calculated. Oxy, Deoxy, THb, and StO_2_ data are presented as the change from the resting level. Unfortunately, muscle blood circulation data for one participant could not be appropriately obtained; therefore, muscle blood circulation data were available for 11 participants. Our earlier research examined the repeatability of measuring tendon blood circulation (Oxy, Deoxy, THb, and StO_2_) [15, 20].

### 2.4. Heart Rate

Throughout the experiment period, heart rate was measured using Biocom's HRV LIVE ver. 1.0 (Biocom, Inc., Seattle, Wash). The participant's earlobe held the Biocom HRM-02 Pulse Wave Sensor, the photoplethysmography monitor utilized in this investigation. Mean values over a given duration (same as blood circulation data) were calculated and were displayed as the change from the resting level.

### 2.5. Statistical Analysis

The means ± SD are used to report values. Significant differences between the measured variables and the resting level were found using a two-way (condition x time) analysis of variance (ANOVA) with repeated measures. During static stretching, one-way ANOVA was used to detect a significant difference in the measured variables from the resting level. At *p* < 0.05, the F ratios for the main effects and interactions were deemed significant. The Bonferroni post-hoc test was used to identify significant differences between means at *p* < 0.05. Mauchly's sphericity test was used in an ANOVA to evaluate the homogeneity of variance. In cases where the sphericity assumption was broken, the Greenhouse-Geisser correction was used. Pearson product-moment correlations were computed to assess the associations among the measured variables. The level of significance was set at *p* < 0.05.

## 3. Results

**Figure 1:**
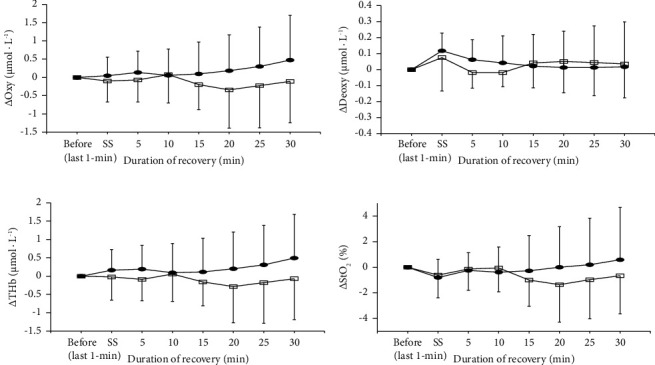
The changes in oxyhemoglobin (Oxy; (a)), deoxyhemoglobin (Deoxy; (b)), total hemoglobin (THb; (c)), and oxygen saturation (StO_2_; (d)) of tendon before, during stretching (only last 1 min point) and recovery periods for 2 min (open square) and 5 min (closed circle) of static stretching.

**Figure 2:**
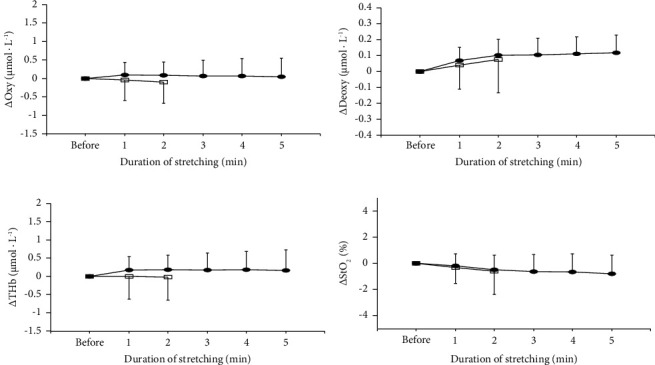
The changes in oxyhemoglobin (Oxy; (a)), deoxyhemoglobin (Deoxy; (b)), total hemoglobin (THb; (c)), and oxygen saturation (StO_2_; (d)) of tendon before and during stretching (every 1 min) for 2 min (open square) and 5 min (closed circle) of static stretching.

The changes in blood circulation of the tendon before, during (only for the last 1 min), and after 2- and 5 min of static stretching are shown in Figure 1. For all measured variables, the effects of condition (*p*=0.164 for Oxy, *p*=0.855 for Deoxy, *p*=0.138 for THb, *p*=0.508 for StO_2_), time (*p*=0.697 for Oxy, *p*=0.343 for Deoxy, *p*=0.722 for THb, *p*=0.530 for StO_2_), and the interaction between condition and time (*p*=0.349 for Oxy, *p*=0.343 for Deoxy, *p*=0.398 for THb, *p*=0.363 for StO_2_) were not significant. The changes in blood circulation of the tendon before and during 2- and 5 min of static stretching are shown in Figure 2. During 2 min of static stretching, the effects of time for all the measured variables were not (*p*=0.629 for Oxy, *p*=0.264 for Deoxy, *p*=0.915 for THb, *p*=0.301 for StO_2_). During 5 min of static stretching, the effect of time for Deoxy was significant (*p*=0.002), although those for Oxy (*p*=0.650), THb (*p*=0.286), and StO_2_ (*p*=0.079) were not.

**Figure 3:**
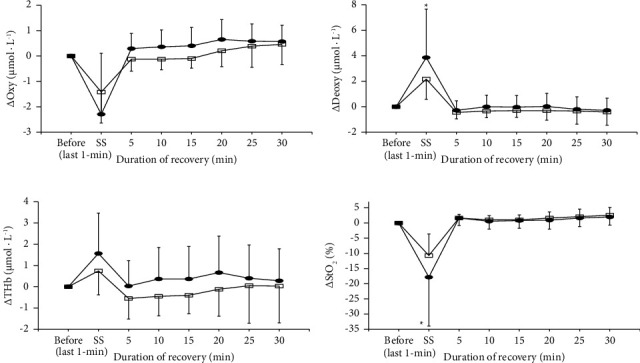
The changes in oxyhemoglobin (Oxy; (a)), deoxyhemoglobin (Deoxy; (b)), total hemoglobin (THb; (c)), and oxygen saturation (StO_2_; (d)) of muscle before, during stretching (only last 1 min point) and recovery periods for 2 min (open square) and 5 min (closed circle) of static stretching. ^*∗*^Significantly different from the resting level (^*∗*^*p* < 0.05).

**Figure 4:**
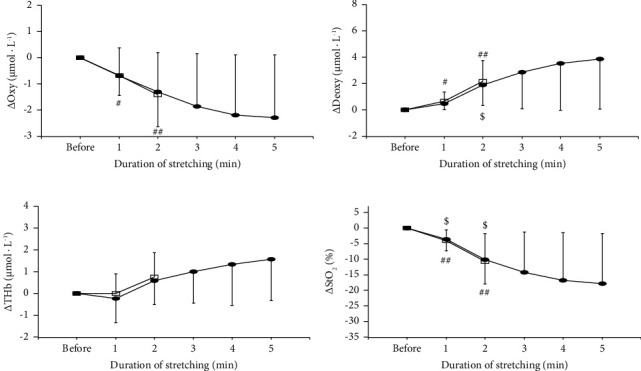
The changes in oxyhemoglobin (Oxy; (a)), deoxyhemoglobin (Deoxy; (b)), total hemoglobin (THb; (c)), and oxygen saturation (StO_2_; (d)) of muscle before and during stretching (every 1 min) for 2 min (open square) and 5 min (closed circle) of static stretching. ^#^Significantly different from the resting level in 2 min of static stretching (^#^*p* < 0.05, ^##^*p* < 0.01). ^$^Significantly different from the resting level in 5 min of static stretching (^$^*p* < 0.05).

The changes in blood circulation of the muscle before, during (only for the last 1 min), and after 2- and 5 min of static stretching are shown in Figure 3. For Oxy, Deoxy, and StO_2_, the effect of time (*p*=0.001 for Oxy, *p* < 0.001 for Deoxy, *p* < 0.001 for StO_2_) was significant, although the effects of condition (*p=*0.378 for Oxy, *p*=0.089 for Deoxy, *p*=0.154 for StO_2_) and the interaction between condition and time (*p*=0.349 for Oxy, *p*=0.343 for Deoxy, *p*=0.363 for StO_2_) were not significant. For THb, the effects of condition (*p*=0.042) and time (*p*=0.023) were significant, although the effect of the interaction between condition and time was not (*p*=0.423). The changes in blood circulation of the muscle before and during 2- and 5 min of static stretching are shown in Figure 4. Deoxy and THb significantly increased during 2 min (*p* < 0.001 for Deoxy, *p*=0.032 for THb) and 5 min (*p*=0.008 for Deoxy, *p*=0.012 for THb) static stretching. Oxy and StO_2_ significantly decreased during 2 min (*p*=0.003 for Oxy, *p* < 0.001 for StO_2_) and 5 min (*p*=0.007 for Oxy, *p*=0.004 for StO_2_) of static stretching.

**Figure 5:**
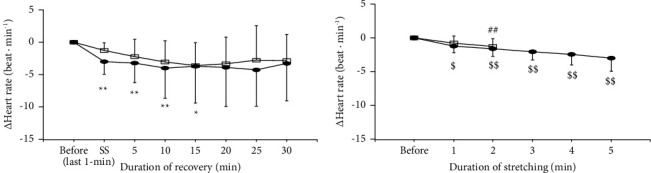
(a) The changes in heart rate before, during stretching (only last 1 min point) and recovery periods for 2 min (open square) and 5 min (closed circle) of static stretching. ^*∗*^Significantly different from the resting level (^*∗*^*p* < 0.05, ^*∗∗*^*p* < 0.01). (b) The changes in heart rate before and during stretching (every 1 min) for 2 min (open square) and 5 min (closed circle) of static stretching. ^#^Significantly different from the resting level in 2 min of static stretching (^##^*p* < 0.01). ^$^Significantly different from the resting level in 5 min of static stretching (^$^*p* < 0.05, ^$$^*p* < 0.01).

**Figure 6:**
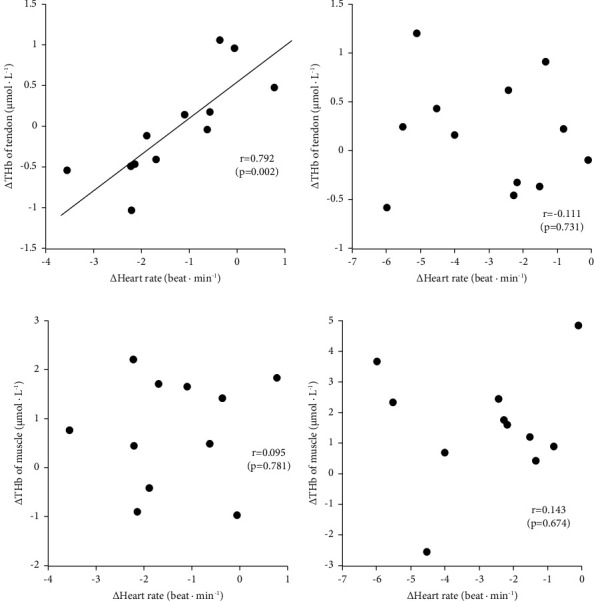
The relationships between changes in the heart rate and blood volume of tendon (a) and (b) and muscle (c) and (d) at the last 1 min in 2 min (a) and (c) and 5 min (b) and (d) of static stretching.

The changes in heart rate before, during (only for the last 1 min), and after 2- and 5 min of static stretching are shown in Figure 5(a). The effect of time was significant (*p*=0.041), although the effects of condition (*p*=0.517) and the interaction between condition and time (*p*=0.775) were not. The changes in heart rate before and during 2- and 5 min of static stretching are shown in Figure 5(b). Heart rate significantly decreased during 2 min (*p*=0.003) and 5 min (*p* < 0.001) of static stretching. The changes in heart rate at the last 1 min during stretching were not associated with those in THb of muscle and tendon, except for the relationship between tendon THb and heart rate in 2 min of static stretching (Figure 6).

## 4. Discussion

The main results of this study were that (1) tendon blood circulation did not change during and after static stretching, (2) muscle Oxy and StO_2_ decreased and Deoxy and THb increased during static stretching but returned immediately upon completion, and (3) the changes in blood volume of tendon and muscle were not associated with those in heart rate (except for the relationship between tendon THb and heart rate in 2 min of static stretching).

Previous studies demonstrated that hyperthermia, acupuncture, hyperbaric oxygen therapy, and repeated eccentric contractions increased blood volume and oxygen saturation of the tendons [10–13]. More recent study has also reported that acupressure stimulation, which imposes a short-axis mechanical load on the tendon, enhances tendon blood circulation [19]. In the present study, we examined whether the tendon blood circulation changes due to static stretching, which was a mechanical stimulus in the long-axis direction of the tendon. Contrary to our expectations, it was found that static stretching did not alter the blood circulation of the tendon. However, during 5 min of static stretching, the tendon Deoxy increased (*p*=0.002), and the tendon StO_2_ tended to decrease (*p*=0.079). These results suggest that blood inflow to the tendon is unchanged (because the tendon Oxy is unchanged), but the metabolisms of the tendon enhance during static stretching.

In the recovery period after static stretching, the reason for the lack of change in tendon blood circulation may be related to insufficient mechanical stimulation of the tendon compared to acupressure and repeated muscle contractions [10, 19]. Although different from stretching, in repeated muscle contractions, tendon blood volume did not change under long-duration conditions in isometric contractions but increased after short-duration (i.e., cyclic) contractions [20]. Considering the previous finding, the tendon blood circulation may change due to dynamic stretching, but not static stretching.

Regarding blood circulation of the muscles, Oxy and StO_2_ decreased, and Deoxy and THb increased during static stretching (Figure 4). These results suggested that both arteries (blood inflow into the muscle) and venous (blood outflow from the muscle) compressed to some extent during static stretching and may cause blood pooling in the capacitance vessels. This result (increased THb during static stretching) was consistent with the results of Kruse et al. [3]. However, several studies reported a decrease in muscle blood volume during static stretching, but the reason for the discrepancy with the present results is unclear. From the result of this study, it may be said that blood circulation within the muscle during static stretching is similar to the situation during low-load blood flow restriction training [21–23]. If so, the combined use of strength training during static stretching may further improve muscle strength and muscle hypertrophy.

After static stretching, there were no significant increases in THb and StO_2_ of muscle as well as tendon. Previous studies reported that blood volume (from near-infrared spectroscopy) and blood flow (from ultrasound Doppler) were significantly higher than at rest until about 10 min after the end of static stretching [2–4, 7]. In this study, since the foot angle at the beginning and end of stretching was changed manually with the attachment, it was impossible to identify the exact beginning and end points of stretching. Therefore, the beginning and end of stretching strictly varied among subjects by a few seconds (less than 10 s at most). Even taking this into account, however, the results of this study differed from the results of these previous studies. Possible reasons for these discrepancies include stretching intensity and age of participants, but the present study cannot address the definite reasons.

In the present study, heart rate significantly decreased during stretching and up to 15 min after stretching. This result is consistent with the results of previous studies, which indicated a decrease in heart rate and an increase in the parasympathetic nervous system with stretching [16–18]. However, the changes in heart rate were not associated with changes in blood circulation in tendon and muscle in this study (except for the relationship between tendon THb and heart rate in 2 min of static stretching). Our previous study on acupuncture stimulation showed a significant negative correlation between heart rate changes and tendon THb during the recovery period, suggesting that the autonomic nervous system is involved in controlling tendon blood circulation after acupuncture stimulation [11]. Conversely, a positive correlation was observed between the changes in tendon blood volume and heart rate during the recovery period with acupressure stimulation, suggesting that increased tendon metabolism due to acupressure stimulation is associated with increased heart rate [19]. The present study found a high positive correlation between the changes in tendon THb and heart rate only in the 2 min of static stretching (Figure 6). This result suggested that static stretching increased tendon metabolism, as in the case of acupressure stimulation. Several studies similarly showed that stretching increased metabolism, including oxygen consumption, use of glycogen, and uptake of blood glucose [24–26]. However, we consider this result of the present study to be due to mere chance since no such significant correlation between the two was found at a 2 min time point during the 5 min of static stretching (*r* = −0.099, data not shown). In a future study, we require additional data for clarification.

It is necessary to highlight the limitations of the employed approach in the current investigation. Firstly, control conditions were not set in this study because we confirmed that blood circulation of the Achilles tendon did not change during control conditions in our previous study [11]. Secondly, the stretching employed in this study differed from the usual method (e.g., limb positions, duration, etc.). Since normal stretching is usually done in the standing position, the effects of one's own body weight and gravity may be different from the stretching employed in this study. In addition, static stretching of a given body district for 5 min is rarely sustained in practice. Furthermore, only two static stretching conditions (2 min and 5 min) were included in the present study. However, the results of this study (especially blood circulation in the tendons) revealed the need to examine the results under more extended time conditions. This point will be verified in future studies. Thirdly, every participant should execute static stretching at the same relative ankle angle to their maximum range of motion. On the other hand, we used 27 degrees of dorsiflexion for every individual. Therefore, it is inevitable that each participant had different stretching intensities. Fourthly, the examiner manually changed the ankle angles before and after stretching, so all participants could not start and finish stretching at precisely the same time. Therefore, it was not possible to calculate the measured variables at fine time intervals before and after the start and end of stretching. Fifthly, the present study was performed with a small sample size. Therefore, for results such as those with a significant difference trend in tendon blood circulation, a clear difference may be detected by increasing the sample size.

## 5. Conclusion

These results suggested that static stretching for 2- and 5 min showed no change in tendon blood circulation, although muscle blood circulation during stretching was altered. Furthermore, heart rate decreased significantly during and after stretching, whereas the changes in heart rate were not associated with changes in muscle and tendon blood circulation (except for the relationship between tendon THb and heart rate in 2 min of static stretching). Future studies should examine the effects of extended periods of static stretching and different stretching modes (ballistic, dynamic, and proprioceptive neuromuscular facilitation stretching) on tendon blood circulation.

## Data Availability

The datasets are available from the corresponding author upon request.
